# Multi-Omics Integration: Predicting Progression and Optimizing Clinical Treatment of Hepatocellular Carcinoma Through Malignant-Cell-Related Genes

**DOI:** 10.3390/ijms26136135

**Published:** 2025-06-26

**Authors:** Qianwen Wang, Lingli Cheng, Honglin Yan, Jingping Yuan

**Affiliations:** Department of Pathology, Renmin Hospital of Wuhan University, Wuhan 430060, China; qwen0806@163.com (Q.W.); rm003736@whu.edu.cn (L.C.); honglin@whu.edu.cn (H.Y.)

**Keywords:** hepatocellular carcinoma, single cell, machine learning, malignant cell

## Abstract

Hepatocellular carcinoma (HCC) presents significant intertumoral heterogeneity, complicating prognosis and treatment. To address this, we performed an integrated single-cell RNA-sequencing analysis of HCC specimens using Seurat and identified malignant cells via Infercnv. Through a systematic evaluation of 101 machine learning algorithms used in combination, we developed tumor-cell-specific gene signatures (TCSGs) that demonstrated strong predictive performance, with area under the curve (AUC) values ranging from 0.72 to 0.74 in independent validation cohorts. Risk stratification based on these signatures revealed distinct therapeutic vulnerabilities: high-risk patients showed increased sensitivity to sorafenib, while low-risk patients exhibited enhanced responses to immunotherapy and transarterial chemoembolization (TACE). Pharmacogenomic analysis with Oncopredict identified four chemotherapeutic agents, including sapitinib and dinaciclib, with risk-dependent efficacy patterns. Furthermore, CRISPR/Cas9-dependency screening prioritized SRSF7 as essential for HCC cell survival, a finding confirmed by the identification of protein-level overexpression in tumors via immunohistochemistry. This multi-omics framework bridges single-cell characterization to clinical decision-making, offering a clinically actionable prognostic system that can be used to optimize therapeutic selection in HCC management.

## 1. Introduction

Hepatocellular carcinoma (HCC), which accounts for 85–90% of primary liver cancer cases, ranks as the third-most-common malignant tumor globally [1,2,3]. Epidemiological data indicate that approximately 840,000 new cases of HCC are diagnosed annually worldwide, leading to at least 780,000 deaths [1]. Current clinical treatment strategies encompass surgical resection, liver transplantation, and local radiotherapy combined with systemic chemotherapy [2]. However, due to the subtle symptoms of the disease in its early stages, most patients have already advanced to later stages by the time of diagnosis, missing the optimal window for treatment. Although the serum alpha-fetoprotein (AFP) test is routinely used for risk stratification of hepatocellular carcinoma [4], its sensitivity and specificity are significantly limited, making it difficult to provide accurate diagnosis and support treatment using this test. Therefore, the development of novel biomarker combinations to optimize early diagnosis, prognostic stratification, and individualized treatment decisions has become a key research direction for work aiming to improve patient survival outcomes.

Current prognostic models, which are primarily derived from bulk transcriptomic or clinicopathological data [5,6,7], fail to distinguish the contributions of malignant cells from the tumor microenvironment (TME), limiting their clinical utility. Unlike traditional bulk-RNAseq, single-cell RNA-seq (scRNA-seq) can acquire transcriptome data for each cell at an unprecedented resolution [8]. Recent studies have revealed complex intra-tumor heterogeneity in the HCC microenvironment and identified various new cell subpopulations using scRNA-seq. Ma and Zheng used single-cell sequencing to map single cells in hepatocellular carcinoma and to resolve the heterogeneity of infiltrating T-cells in hepatocellular carcinoma tissues, respectively [9,10]. Nevertheless, further investigation is required to fully elucidate the gene-expression characteristics of tumor cells in HCC.

Copy-number variation (CNV) is the primary form of variation caused by genomic rearrangements. CNV can result in gene amplification (copy-number gain) and deletion (copy-number loss), which may correspond to an increase or decrease, respectively, in the production of the RNA and protein encoded by a gene [11]. CNV has been described as a hallmark of all cancers, and several studies have demonstrated that copy-number changes in specific genes, particularly oncogenes and tumor suppressor genes, play a role by affecting the expression levels of these genes. These changes play a role in tumorigenesis and tumor development, progression, drug sensitivity, and resistance by affecting the expression levels of these genes [12,13,14,15].

Therefore, this study aims to process scRNA-seq datasets to differentiate tumor cells from epithelial cells by inferring copy-number variations (CNVs). Subsequently, multiple machine learning algorithms were employed to construct prognostic models related to tumor cells, predicting liver cancer patients’ prognosis and their response to clinical treatments. Beyond prognostication, we explored the model’s ability to predict therapeutic sensitivities, effectively bridging the gap between molecular profiling and clinical decision-making. This work not only advances HCC risk stratification but also provides actionable insights for tailoring therapies to tumor-cell-driven vulnerabilities.

## 2. Results

### 2.1. Processing Single-Cell Data

**Figure 1:**
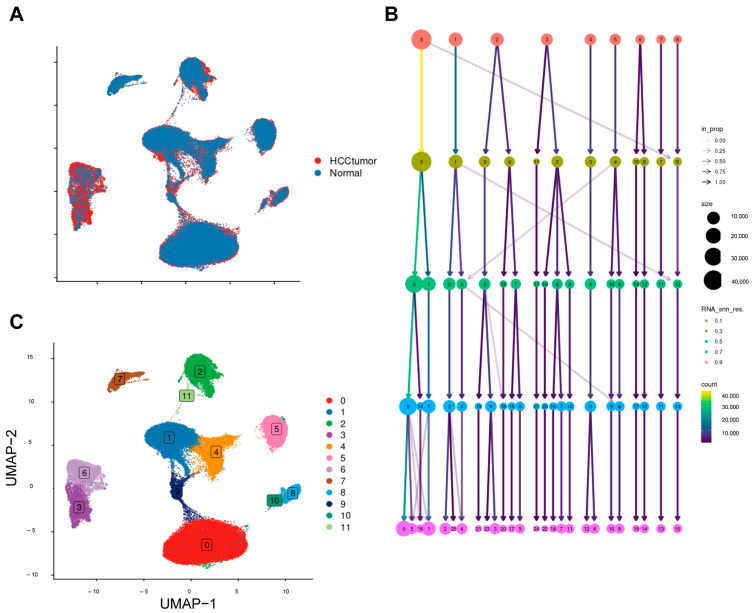
Creation of single−cell objects. (**A**) UMAP plot demonstrating the effect of harmony integration; (**B**) clustering tree demonstrating cellular clustering at different resolutions; (**C**) cellular clustering at resolution = 0.3.

A total of 110,658 cells were obtained after creation of the single-cell object, and 98,854 cells remained after quality control, followed by removal of batch effects using harmony (Figure 1A). The choice of resolution is crucial in the processing of single-cell data; too low or too high a resolution can result in cell populations being over-merged or over-segmented. Based on the results of the clustering tree (Figure 1B), we determined the resolution to be 0.3 (RESOLUTION = 0.3), and from this, we obtained 12 cell subpopulations (Figure 1C).

### 2.2. Annotations for Cell Types

**Figure 2:**
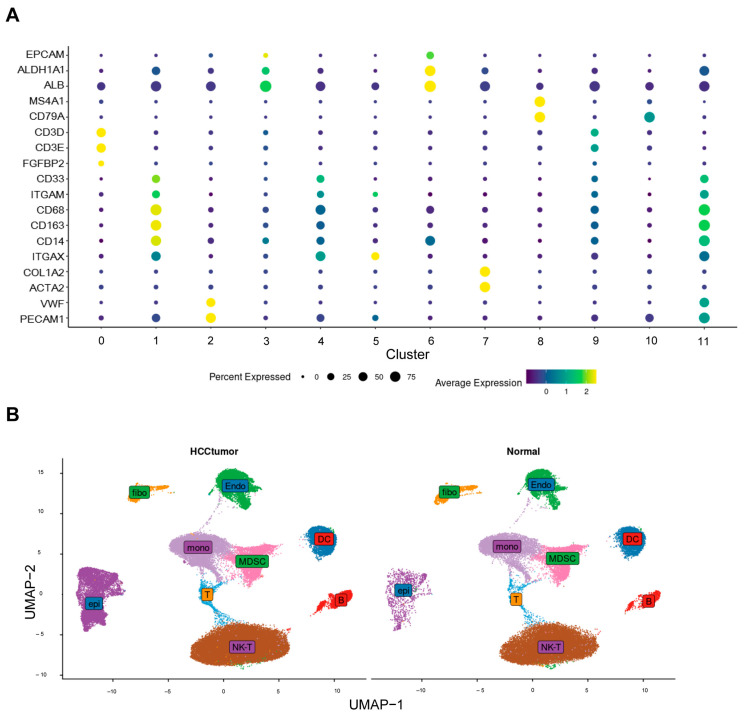
Annotation of cell clusters. (**A**) Expression of classical cell markers between clusters. (**B**) Cell distribution between tumor tissue and normal tissue.

We defined cluster 9 as T cells (CD3D, CD3E); cluster 0 as NKT cells (CD3D, CD3E, and FGFBP2); cluster 2 as endothelial cells (VWF and PECAM1); cluster 4 as MDSC cells (CD33 and ITGAM); clusters 8 and 10 as B cells (MS4A1 and CD79A); and clusters 1 and 11 as monocytes (CD68, CD163, and CD14). Clusters 3 and 6 were defined as epithelial cells (EPCAM, ALDH1A1, and ALB), cluster 7 as fibroblasts (COL1A2 and ACTA2), and cluster 5 as dendritic cells (ITGAX) [16,17]. Dot plots (Figure 2A) were used to illustrate the expression of marker genes, and UMAP plots (Figure 2B) were used to demonstrate the distributions of these cell populations in HCC and normal tissues.

### 2.3. InferCNV

**Figure 3:**
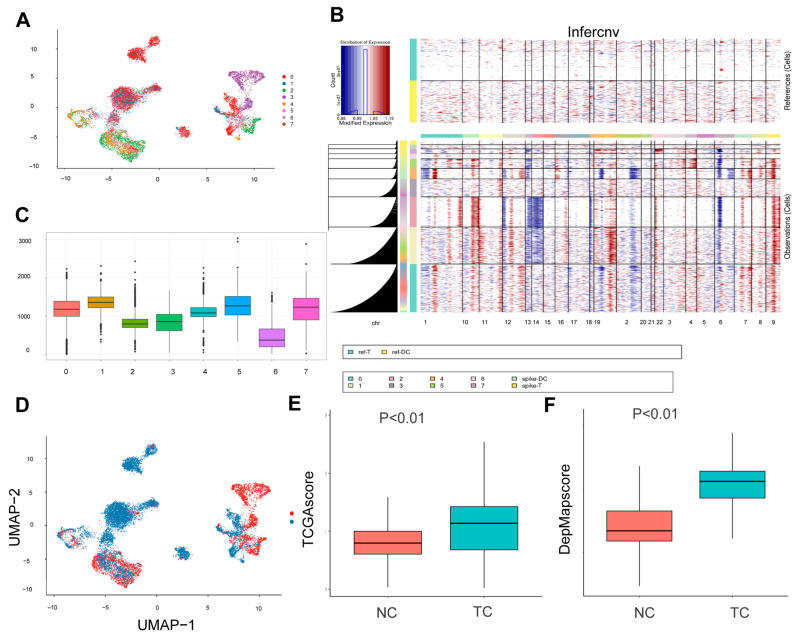
Infercnv identifies tumor cells. (**A**) Epithelial cell subpopulation results. (**B**) Heat map of Infercnv results. Genomic amplification in red and genomic deletions in blue. (**C**) Box line plot showing the CNV scores of different epithelial cell subpopulations. (**D**) UMAP plot showing the distributions of normal epithelial cells and tumor cells. (**E**) Difference analysis of malignancy scores (malignant gene set from TCGA) of tumor cells and normal cells. (**F**) Difference analysis of malignancy scores (malignant gene set from DepMap). Red indicates normal cells, and green indicates tumor cells.

Subsequently, a total of 11,789 epithelial cells were extracted and re-clustered into eight distinct subpopulations (Figure 3A). The Infercnv algorithm [18] was then used to infer the identity of possible tumor cells using T and DC cells as reference cells (Figure 3B). It is worth noting that although T cells and DCs are generally considered to exhibit genomic stability, minor CNV-like signals may still emerge due to transcriptional noise or stress-induced fluctuations in gene expression, as previously reported [19,20]. Clusters 0, 1, 4, 5, and 7 were found to have significantly higher CNV scores compared to the other cell clusters (Figure 3C), thereby indicating their potential identities as tumor cells (Figure 3D). The top 100 genes with elevated expression (ranked by LogFC) in hepatocellular carcinoma tissues were obtained from GEPIA (Table S1), and the top 100 genes associated with the survival of hepatocellular carcinoma cells (ranked by Gene effect) were obtained from DepMap (a genome-wide screening database) (Table S2). Scoring of tumor and normal cells revealed that malignancy scores were significantly higher in tumor cells than in normal cells (Figure 3E,F).

### 2.4. Machine Learning to Build Models

**Figure 4:**
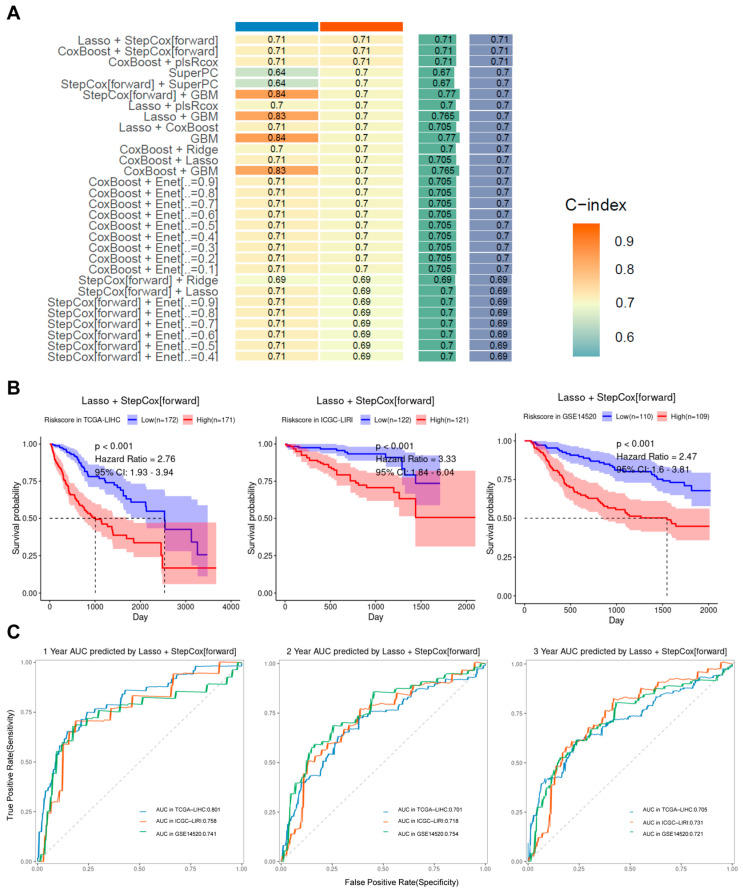
Machine learning-constructed models. (**A**) Heatmap of C-index scores for 101 machine learning models (only results for training sets with C-index > 0.70 are shown). (**B**) Survival analysis of patients in high–- and low–-risk groups. (**C**) Time-dependent curves demonstrating the ability of risk scores to predict patient survival.

To enhance the prognostic power of tumor-cell-related genes in hepatocellular carcinoma (HCC), we initially evaluated 101 combinations of machine learning and survival-modeling pipelines using the mime R package. Among them, Lasso regression followed by forward stepwise Cox proportional hazards modeling (Lasso + StepCox[forward]) achieved the highest concordance index (C-index) on the validation dataset (Figure 4A). To rigorously evaluate its generalization ability and minimize the risk of overfitting, we conducted a formally nested 10-fold cross-validation procedure: the inner loop performed feature selection and hyperparameter tuning, while the outer loop was used to generate unbiased performance estimates. The final model achieved a mean C-index of 0.633 ± 0.092 on outer test folds, demonstrating acceptable robustness and predictive utility. In addition, a learning curve was generated based on the sizes pf the training subsets (10% to 100%) during outer-loop cross-validation. The curve showed a consistent increase in performance with training-sample size and a relatively small gap between training and validation C-index, indicating that overfitting in the model is not a severe problem (Figure S1A). This supports the stability and scalability of the selected Lasso + StepCox model. Consequently, a panel of 15 genes—namely SRSF7, NQO1, APCS, NUPR1, SCARB1, DAB2, ANG, PPP2R1B, C1S, SERPINE1, GADD45G, ITIH1, PDK4, HSP90AA1, and SAT1—was selected for further investigation and identified as model-building genes. Subsequently, hepatocellular carcinoma patients were categorized into a low-risk group and a high-risk group according to their risk scores, and it was found that hepatocellular carcinoma patients in the low-risk group had better survival outcomes than those in the high-risk group in the databases of the three independent sources (Figure 4B). The time-dependent ROC curves also demonstrated that TIRGs were a reliable predictor of survival, with 1-, 2-, and 3-year areas under the curve (AUC) being ≥ 0.70 (Figure 4C). It is noteworthy that the model constructed in this study demonstrated significant superiority over other published models (Table S3), as evidenced by a considerably higher C-index and superior performance on both the training and validation sets (Figure S1B).

### 2.5. Risk Scores Correlate Strongly with Clinical Characteristics

**Figure 5:**
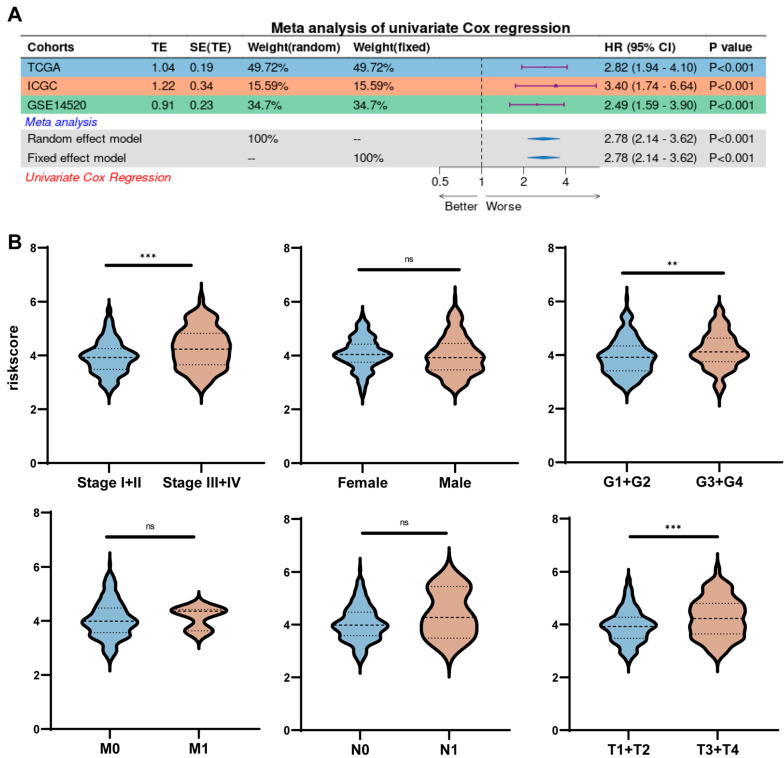
Risk scores were strongly correlated with clinicopathological characteristics. (**A**) Forest plot illustrating the results of univariate Cox regression analysis. (**B**) Box–and–line plot illustrating the relationship between pathological characteristics and risk scores. ** *p* < 0.01, *** *p* < 0.001, ns: not significant.

Univariate COX analysis showed that risk score was an independent prognostic factor for HCC in all three datasets (*p* < 0.001) (Figure 5A). To further understand the relationship between risk score and clinical characteristics of HCC, we analyzed risk score together with other clinical characteristics of HCC patients. It was found that risk scores were higher for patients with worse pathological staging and grading of tumors and larger tumor volume, while there was no difference in other clinical characteristics (Figure 5B).

### 2.6. Risk Scores in Combination with Other Clinicopathological Characteristics

**Figure 6:**
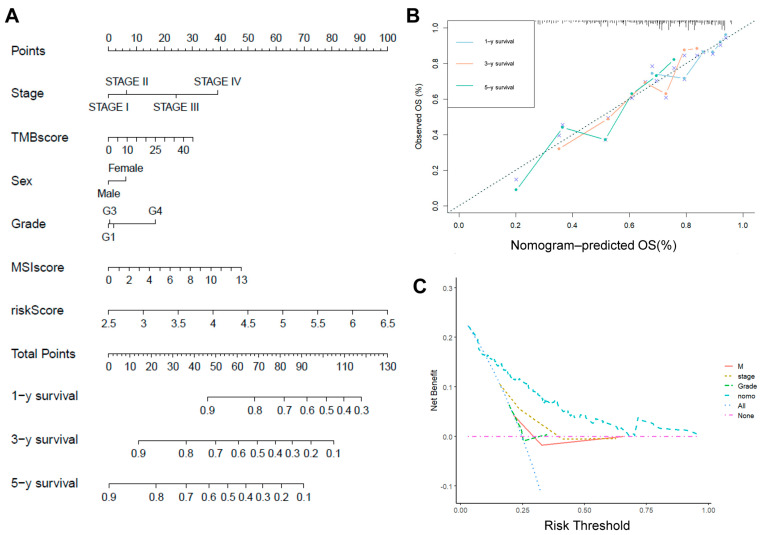
Risk scores were analyzed in conjunction with other clinicopathological features. (**A**) Column line graph analyzing the clinical predictive power of risk scores. (**B**) Calibration curve testing the predictive power of risk models. (**C**) DCA assessing the clinical diagnostic power of risk scores.

A new column–linear graphical model was developed by combining risk scores and clinical characteristics to predict the 1-, 3- and 5-year survival probabilities of HCC patients (Figure 6A). The calibration curves showed that the 1, 3, and 5-year OS rates predicted by the nomograms were close to the actual OS rates (Figure 6B), and the decision curve analysis (DCA) showed that the risk scores, in combination with the other clinical characteristics, outperformed the individual clinical characteristics in terms of predictivity (Figure 6C). Previously constructed prognostic models often lacked credibility due to the lack of validation on large data samples of clinical proteins. To increase the credibility of the model, we downloaded HCC proteomic data and the corresponding clinical profiles from CPTAC and performed joint analyses with clinical features. The results were consistent with those obtained at the mRNA level, and the predictive effect of risk scores in combination with other clinical features was better than that of individual clinical features (Figure S2A–C).

### 2.7. Assessing the Relationship Between Risk Scores and Common Treatments

**Figure 7:**
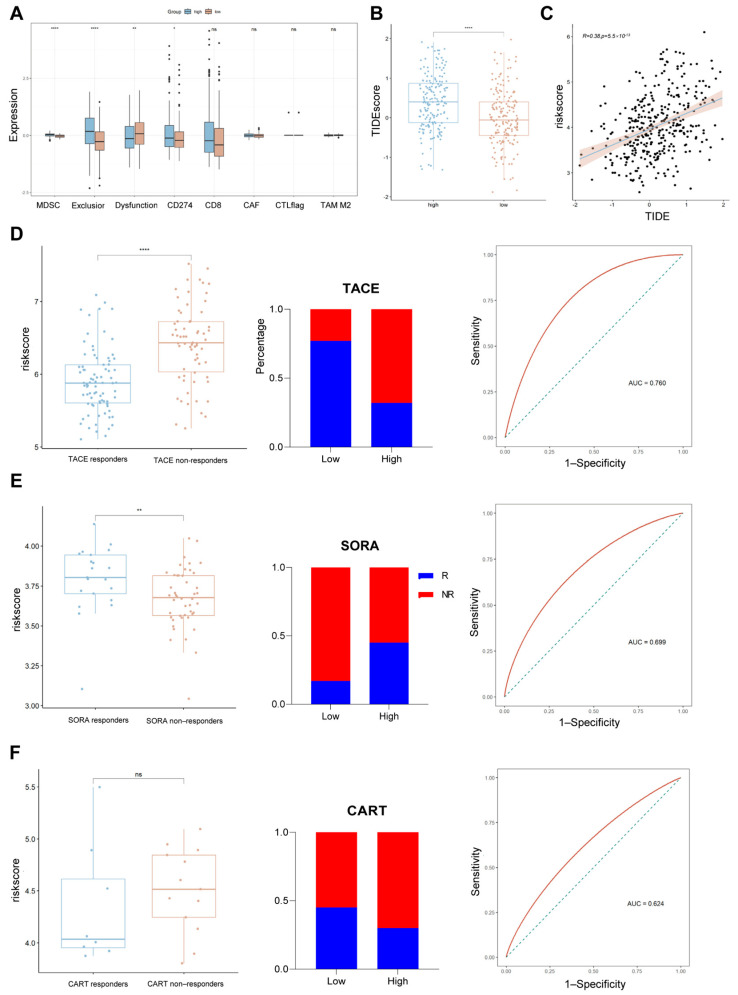
Relationship between risk scores and commonly used therapies for HCC. (**A**) Differences in immune function between risk groups. (**B**) Box line plot demonstrating TIDEScore between risk groups. (**C**) Positive correlation between risk scores and TIDEScore. (**D**–**F**) Relationship between risk scores and responses to TACE, SORA, and CART therapies. From left to right: risk scores for patients in different response groups, bar graphs showing the distributions of responders and non-responders in high- and low-risk groups, and ROC curves demonstrating the ability of risk scores to distinguish between responders and non-responders. * *p* < 0.05, ** *p* < 0.01, **** *p* < 0.0001, and ns means not significant.

Surgical resection is the first-line treatment for early-stage HCC and is the most widely used treatment in this stage [21]. However, the majority of patients with HCC are in the intermediate and advanced stages at the time of diagnosis. In view of this situation, to improve the practical value of risk scores, this study investigated the intrinsic association between risk scores and commonly used treatments for intermediate- and advanced-stage HCC (chemotherapy, TACE, CART, and immunotherapy) [22,23,24]. We found that the high-risk group had higher immune-dysfunction scores and TIDE scores (Figure 7A,B) and that risk scores were positively correlated with TIDE scores (Figure 7C), suggesting that patients in the high-risk group were less likely to respond to immunotherapy and had a worse prognosis. In addition, sorafenib treatment may be more suitable for patients in the high-risk group (Figure 7E), whereas TACE treatment may be more suitable for those in the low-risk group (Figure 7D), and the risk score may more accurately discriminate between responders and non-responders to sorafenib and TACE. Unfortunately, there was no significant difference in risk scores between CART responders and non-responders (Figure 7F).

### 2.8. Assessing the Relationship Between Risk Scores and Drug Sensitivity

**Figure 8:**
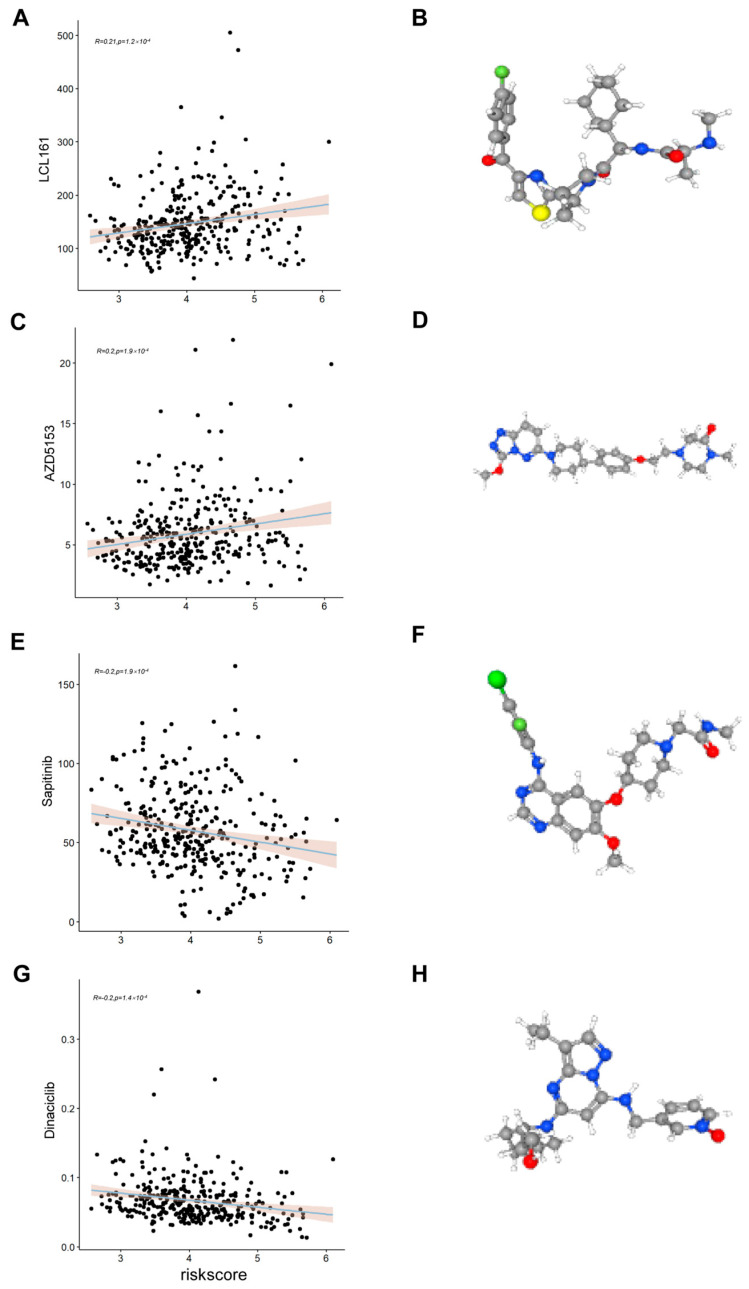
Predicting drug sensitivity. (**A**,**C**,**E**,**G**) Scatter plots of the correlation of risk scores with LC161, AZD5153, sapitinib, and dinaciclib. (**B**,**D**,**F**,**H**) 3D structural maps of LC161, AZD5153, sapitinib, and Dinaciclib from PubChem. Carbon atoms are shown in gray, hydrogen in white, oxygen in red, nitrogen in blue, sulfur in yellow, phosphorus in orange, chlorine in green, bromine in dark red, iodine in violet, and fluorine in light green.

To identify other chemotherapeutics for which responsiveness might be associated with risk scores, we evaluated the IC50s of all samples in TCGA-LIHC for 192 chemotherapeutics using GDSC2 as a training set and screened chemotherapeutics from them for correlation with risk scores (*p* < 0.01, |R| > 0.2). We found that the IC50 values of HCC patients for LC161 and AZD5153 were positively correlated with risk score (Figure 8A,C) and that the IC50 values of sapitinib and dinaciclib were negatively correlated with risk score (Figure 8E,G), suggesting that sapitinib and dinaciclib may be more suitable for use in patients in the high-risk group, while patients in the low-risk group may be potentially responsive to LC161 and AZD5153. In addition, the 3D structures of the four chemotherapeutics were visualized using PubChem (Figure 8B,D,F,H). It is important to note that this extrapolation needs to be validated by subsequent pharmacological studies.

### 2.9. SRSF7 Is Highly Expressed in Hepatocellular Carcinoma Tissues and Is Associated with a Poor Prognosis

**Figure 9:**
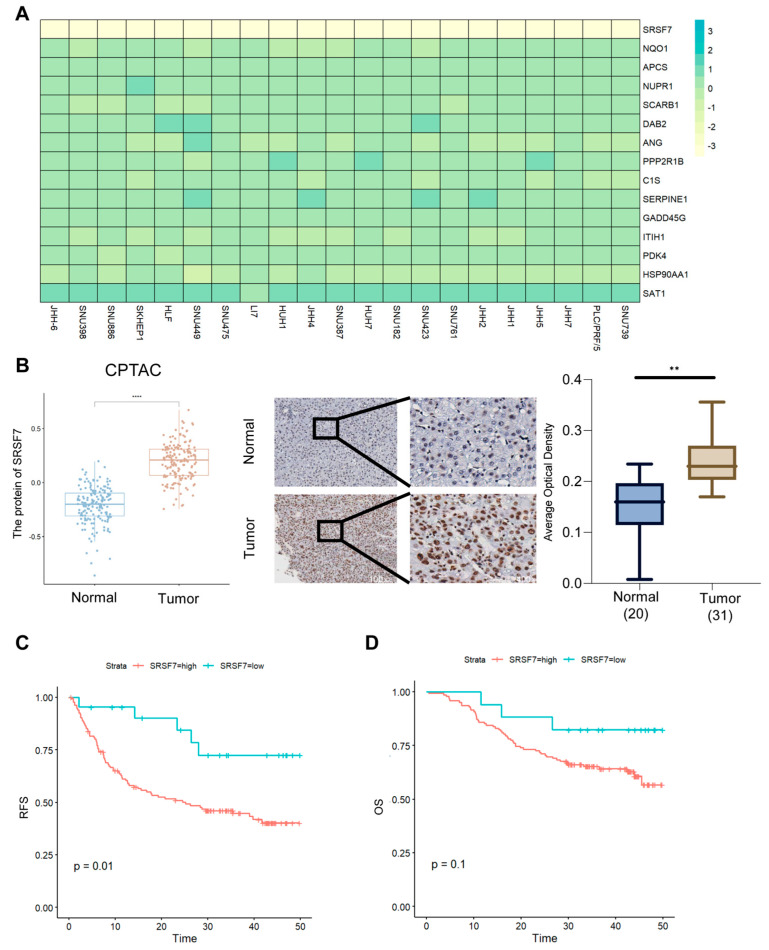
Poor prognosis of hepatocellular carcinoma in patients with high expression of SRSF7. (**A**) Gene=effect heatmap of prognostic genes in hepatocellular carcinoma cells from DepMap. (**B**) SRSF7 protein expression from the CPTAC (protein database). (**C**) Expression of SRSF7 in clinical hepatocellular carcinomas and normal tissue samples and the corresponding statistical results. (**D**) Recurrence-free survival (RFS) and overall survival (OS) in high- vs. low-SRSF7-expression groups. ** *p* < 0.01, **** *p* < 0.0001.

Data from DepMap (genome-wide screening databases) indicated that SRSF7 was most strongly associated with HCC cell survival among all model constructs and that HCC cell survival was most inhibited after SRSF7 knockdown (Figure 9A). We found that the SRSF7 protein was highly expressed in liver cancer tissues using protein databases (CPTAC) and immunohistochemistry from clinical samples (Figure 9B) and that liver cancer patients with high SRSF7 protein expression had worse progression-free survival (Figure 9C). However, there was no significant difference in disease-recurrence-free survival between hepatocellular carcinoma patients in the high- and low-SRSF7-expression groups (Figure 9D). Tumor cells with high and low expression of SRSF7 showed significant differences in apoptosis signaling pathways, in glycine, serine, threonine metabolism, and in glycerophospholipid metabolism pathways (Figure S3A–C). In addition, we knocked down the expression of SRSF7 in HepG2 (Figure S4A,B) and found that the viability of hepatocellular carcinoma cells was significantly reduced (Figure S4C–E).

## 3. Discussion

Hepatocellular carcinoma (HCC) has been shown to originate from epithelial cell populations and is composed of tumor cells, basement membrane, and surrounding stroma [25,26,27]. Its development has been found to be closely associated with specific gene mutations, new tumor subpopulations, and oncogenic pathways [28]. Tumor cells have been demonstrated to play a pivotal role in the remodeling of the tumor microenvironment, exerting a significant influence on disease progression, patient prognosis, and response to chemotherapy. A comprehensive understanding of the intricate interactions between tumor cells and the TME and of how they function in conjunction with the mechanisms of chemotherapy resistance is paramount for the development of more efficacious therapeutic strategies for HCC.

In this study, a prognostic model was constructed for the purpose of predicting the outcomes of patients suffering from hepatocellular carcinoma (HCC). The model was constructed using data from single-cell transcriptomic analysis in combination with multi-omics analysis. This approach provides a new perspective on the assessment of prognosis and the development of individualized treatment strategies for HCC patients. The model was constructed using 101 machine learning algorithms, which significantly improved its stability and generalization. In comparison with previous models based on bulk transcriptomes, the present model focuses on tumor-cell-specific driver genes and avoids interference from confounding signals in the tumor microenvironment, thus achieving a significant advantage in prediction of medium- and long-term survival. In addition, the validation of the model in an independent proteomics dataset further supports its biological reliability, especially given the high expression of the key gene SRSF7 in hepatocellular carcinoma at the protein level and its association with poor prognosis, suggesting the potential role of the model in HCC progression.

The prognostic model constructed in this study incorporated 15 tumor-cell-specific genes (SRSF7, NQO1, APCS, NUPR1, SCARB1, DAB2, ANG, PPP2R1B, C1S, SERPINE1, GADD45G, ITIH1, PDK4, HSP90AA1, SAT1) associated with progression and treatment resistance. SRSF7 is a core factor in the regulation of RNA splicing that affects the plasticity and metastatic potential of tumor cells through selective splicing. High expression of SRSF7 in HCC promotes cell proliferation through activation of the MAPK/ERK pathway and correlates with poor prognosis. Its splicing activity may regulate the expression of isoforms of immune-checkpoint molecules (e.g., PD-L1) and indirectly affect immune escape [29,30]. NQO1 promotes hepatocellular carcinoma invasion and metastasis through enhancement of ERK-NRF2. APCS has been demonstrated in pancreatic cancer to promote fibrosis through the TGF-β signaling pathway, suggesting it may play a role in stromal remodeling in HCC. The expression level of NUPR1 was significantly correlated with vascular infiltration and advanced TNM staging, and its small-molecule inhibitor, ZZW-115, significantly inhibited hepatocellular carcinoma progression [31]. SCARB1 promotes reprogramming of lipid metabolism in tumor cells by mediating high-density lipoprotein (HDL) uptake [32]. DAB2, as a negative regulator of the transforming growth factor β (TGF-β) signaling pathway, is often downregulated in HCC due to promoter methylation and epigenetic modifications [33]. ANG promotes HCC progression by inducing angiogenesis and rRNA transcription. Serum ANG levels are positively correlated with microvessel density (MVD) and postoperative recurrence rate [34]. GADD45G acts as a negative regulator of the Jak-Stat3 pathway and inhibits HCC by inducing cellular senescence [35]; Deficient PDK4 expression promotes hepatocellular carcinoma cell proliferation, tumorigenicity, motility, and invasiveness [36]. HSP90AA1 is associated with poor prognosis in hepatocellular carcinoma and contributes to tumorigenesis and chemoresistance [37].

Furthermore, the ability of this model to provide risk stratification provides a significant foundation for individualized treatment. The sensitivity of low-risk patients to immune-checkpoint inhibitors and TACE therapy may be related to their immune-activation microenvironment (high expression of immunomodulatory genes such as SCARB1 and ITIH1), whereas the response of high-risk patients to sorafenib may depend on the regulation of pro-apoptotic genes such as NUPR1 and GADD45G. Furthermore, a drug-sensitivity analysis revealed an association between risk scores and sensitivity to chemotherapeutic drugs, suggesting that the model may provide a reference for the selection of chemotherapy regimens.

Nevertheless, the present study is not without its limitations. Firstly, the construction of the model is dependent on retrospective public datasets, and its clinical application will require validation in prospective, real-world patient cohorts. Secondly, although the gene SRSF7 was identified as a key risk gene, its precise biological function and regulatory mechanism in hepatocellular carcinoma (HCC) remain unclear and warrant further in-depth investigation through functional experiments. Thirdly, the current analysis focuses primarily on transcriptomic features of tumor cells, while the dynamic interactions between tumor cells and their surrounding microenvironment were not fully considered. Future studies integrating spatial transcriptomics are needed to capture the spatial heterogeneity and cellular context more comprehensively and may thus further improve model interpretability and performance. Lastly, our experimental validation was limited to a single HCC cell line (HepG2), which, while widely used, may not fully capture the biological diversity of hepatocellular carcinoma. Validation in additional HCC cell lines or primary tumor cells will strengthen the generalizability of our findings.

In conclusion, the prognostic model based on tumor-cell-related genes constructed in this study provides a reliable tool for predicting the survival of patients with HCC. Furthermore, it establishes the foundation for precise stratification of immunotherapy, targeted therapy, and chemotherapy. Future studies can further promote the translation of this model into clinical practice through multicenter cooperation and exploration of mechanisms.

## 4. Materials and Methods

### 4.1. Data Sources and Processing

TCGA-LIHC and ICGC-LIRI were obtained from UCSC Xena (http://xena.ucsc.edu (accessed on 1 June 2024)). GSE14520, GSE100797, GSE104580, GSE109211, and GSE202642 were obtained from GEO (https://www.ncbi.nlm.nih.gov/geo (accessed on 1 June 2024)). Proteomic data from hepatocellular carcinoma were obtained from CPTAC (https://gdc.cancer.gov (accessed on 1 June 2024)).

### 4.2. Processing of Single-Cell Data

The ‘Seurat’ R package (version 4.0, Satija Lab, New York, NY, USA) was employed for the processing of single-cell data. The CreateSeuratObject (min.cells = 5&min.features = 300) was used to read and create a single-cell object, with cells containing more than 20% mitochondrial genes being discarded. The ‘Harmony’ R package(version 1.2.0, Yosef Lab, San Francisco, CA, USA) was then employed for the merging and integration of data. Principal Component Analysis (PCA) was performed by setting the number of principal components (PCs) to 20. The ‘clustree’R package (version 0.5.1, Jacqueline Buros Novik, Cambridge, MA, USA) was then used to further reduce the dimensionality of the data and visualize it using UMAP after the resolution had been determined.

### 4.3. Identifying Tumor Cells

CNVs were analyzed using the ‘InferCNV’ R package (version 1.16.0; https://github.com/broadinstitute/inferCNV). The reference cell types were set to be T-cells and DC-cells, and 500 reference cells were randomly selected as control cells from the epithelial cells. The tumor cells among the epithelial cells were identified based on their CNV scores. The top 100 genes with up-regulated expression in liver cancer tissues were obtained from GEPIA, and the top 100 genes with minimal gene effects were obtained from DepMap. Genome-wide CRISPR/Cas9 proliferation screening data for HCC Cell Lines were obtained from DepMap [38,39,40] (https://depmap.org/portal (accessed on 1 June 2024)). The gene-effect score was used to represent the changes in cell growth after a gene of interest was knocked out. A score of 0 indicates that a gene is not essential for cell growth; correspondingly, a score of −1 is comparable to the median of all pan-essential genes. The lower the score, the more likely the gene is an oncogene. The higher the score, the more likely the gene is a tumor-suppressor gene. Malignant genes were scored using ‘AUCell’ R package (version 1.24.0, Sara Aibar, Barcelona, Spain).

### 4.4. Calculation of Malignancy Scores

To quantify the malignancy potential of tumor-like cells identified by inferCNV, we constructed two gene sets: (1) the top 100 overexpressed genes in HCC tissues, ranked by log2 fold-change from GEPIA analysis, and (2) the top 100 essential genes for HCC cell survival, ranked by gene-effect scores from the DepMap database. The enrichment scores for each cell were calculated using the GSVA (Gene Set Variation Analysis) method implemented in the GSVA R package. GSVA R package (version 1.50.0, Bioconductor), implementing the algorithm originally proposed by Sonja Hänzelmann (Heidelberg, Germany).These scores were interpreted as malignancy scores and compared between tumor clusters.

### 4.5. Machine Learning

The ‘Mime’ package was employed to construct prognostic or diagnostic models from the transcriptome [41], integrating 101 machine learning algorithms. The machine learning was performed using the ‘Mime’ package with TCGA-LIHC (training set) and ICGC-LIRI (validation set) and GSE14520 (validation set).

Risk Score=(0.3366×SRSF7)+(0.0480×NQO1)+(−0.0244×APCS)+(0.0445×NUPR1)+(0.2954×SCARB1)+(0.1260×DAB2)+(−0.0314×ANG)+(−0.0838×PPP2R1B)+(−0.0822×C1S)+(0.0430×SERPINE1)+(−0.0334×GADD45G)+(−0.0913×ITIH1)+(0.0115×PDK4)+(0.2080×HSP90AA1)+(−0.1134×SAT1)

### 4.6. Evaluating and Comparing Models

The ‘Survival’ package and the ‘TimeROC’ package were used to plot K–M survival curves and time-dependent ROC curves. The ‘Rms’ package and the ‘ggDCA’ package were used to plot nomograms and DCA decision curves.

### 4.7. Immunohistochemistry

Immunohistochemical analysis was conducted on formalin-fixed paraffin-embedded tissue specimens from 31 HCC patients who underwent surgical resection without radiotherapy or chemotherapy and 20 healthy patients; the samples were collected at Wuhan University People’s Hospital in 2022–2024. Informed consent was obtained from all patients, and the study was approved by the Ethics Committee of Wuhan University People’s Hospital. Tissue wax blocks were cut into 4 mm sections and stained by IHC using the EnVision method. The SRSF7 antibody was purchased from Abcam (ab137247) at a dilution of 1:2000, and all procedures were performed according to the manufacturer’s instructions.

### 4.8. Cell Experiments

HepG2 were obtained from the Cell Collection Committee of Chinese Academy of Sciences (Shanghai, China). HepG2 were cultured using complete medium (90% DMEM and 10% FBS). Cell transfection: experiments were performed using siRNA (Gene Pharma Co., Ltd., Shanghai, China) as well as Lip3000 kit (Invitrogen, Carlsbad, CA, USA). lip3000: siRNA was added to the cells at a ratio of 1:1, and the cells were incubated at 37 °C for 24–48 h for other experiments.

CCK-8 assay: Cells were seeded into 96-well plates at a density of 5000 cells per well in 100 μL of cell suspension and incubated at 37 °C. At 0, 24, 48, and 72 h, 10 μL of CCK-8 reagent (APExBIO, Houston, TX, USA) was added to each well, followed by incubation for 2–4 h. The absorbance at 450 nm was then measured using a microplate reader (Thermo Fisher Scientific, Waltham, MA, USA).

Cell-colonization assay: Cells were spread in six-well plates (2000 cells per well), with the medium changed every 3–5 d, and incubated for 10–14 d.

### 4.9. RT-PCR

Total RNA was extracted using the Total RNA Kit I (Omega Bio-Tek, Norcross, GA, USA), and complementary DNA (cDNA) was synthesized using a reverse-transcription kit (Thermo Fisher Scientific, Waltham, MA, EUA). Afterwards, cDNA samples were subjected to quantitative real-time PCR on a CFX96 system (Bio-Rad, Hercules, CA, USA). The siRNA and primer sequences are shown in Table S4.

### 4.10. Western Blot

Equal amounts of protein extracts were separated by SDS-PAGE and transferred to a PVDF membrane, then incubated with the internal reference antibody β-actin antibody (1:50,000, Cat# 66009-1-Ig, Proteintech, Rosemont, IL, USA) and the target antibody SRSF7 antibody (1:1000, Cat# ab72616, Abcam, Cambridge, UK), with an automated Chemi Scope imaging system (Clinx Science Instruments, Shanghai, China) used for exposure.

### 4.11. Data Analysis

Statistical analyses were performed using GraphPad Prism (9.0) and R software (4.4.1) using an unpaired *t*-test and kruskal.test. * *p* < 0.05, ** *p* < 0.01, *** *p* < 0.001, **** *p* < 0.0001.

## 5. Conclusions

The prognostic model based on tumor-cell-associated genes that was constructed was capable of accurately predicting the survival of patients with hepatocellular carcinoma, thus providing a potential basis for the stratification of clinical treatment of HCC patients.

## Data Availability

The datasets analyzed in this study were obtained from publicly available repositories, as described in the Methods section. The code used for model training, including nested cross-validation and learning curve analysis, is publicly available at https://github.com/shanchangziya/learning-curves-cindex. Other relevant data and scripts supporting the findings of this study are available from the corresponding author upon reasonable request.

## Supplementary Materials

The following supporting information can be downloaded at: https://www.mdpi.com/article/10.3390/ijms26136135/s1.
