# Supplementary material for: Multi-Omics Integration: Predicting Progression and Optimizing Clinical Treatment of Hepatocellular Carcinoma Through Malignant-Cell-Related Genes
Source: Int J Mol Sci. 2025 Jun 26;26(13):6135. doi: 10.3390/ijms26136135 (PMC12249523; doi:10.3390/ijms26136135)
Supplement: Supplementary file 1 [file ijms-26-06135-s001.zip › 修改后的supplementary file/TableS1.docx]

| Gene Symbol | Gene ID | Median (Tumor) | Median (Normal) | Log2(Fold Change) | adjp |
| --- | --- | --- | --- | --- | --- |
| RP11-40C6.2 | ENSG00000219928.2 | 389.91 | 0 | 8.611 | 3.30E-78 |
| GPC3 | ENSG00000147257.13 | 148.158 | 0.92 | 6.28 | 6.38E-59 |
| AKR1B10 | ENSG00000198074.9 | 177.158 | 3.015 | 5.472 | 9.84E-30 |
| UBD | ENSG00000213886.3 | 334.949 | 7.468 | 5.31 | 1.60E-66 |
| MDK | ENSG00000110492.15 | 132.339 | 3.805 | 4.794 | 7.57E-81 |
| MTND4P12 | ENSG00000247627.2 | 39.65 | 0.81 | 4.489 | 5.49E-17 |
| PLVAP | ENSG00000130300.8 | 43.03 | 2.464 | 3.668 | 7.99E-127 |
| OR2I1P | ENSG00000237988.3 | 25.71 | 1.305 | 3.535 | 2.25E-34 |
| PTTG1 | ENSG00000164611.12 | 20.589 | 0.87 | 3.529 | 5.59E-80 |
| ACSL4 | ENSG00000068366.19 | 33.02 | 2.355 | 3.342 | 4.63E-30 |
| PIGY | ENSG00000255072.1 | 8.94 | 0.005 | 3.306 | 6.41E-13 |
| CCL20 | ENSG00000115009.11 | 22.42 | 1.5 | 3.228 | 7.05E-19 |
| CDKN2A | ENSG00000147889.16 | 10.79 | 0.315 | 3.164 | 9.92E-63 |
| UBE2C | ENSG00000175063.16 | 13.24 | 0.6 | 3.154 | 1.56E-68 |
| CCNB1 | ENSG00000134057.14 | 14.8 | 0.9 | 3.056 | 1.24E-71 |
| CDKN3 | ENSG00000100526.19 | 10.96 | 0.45 | 3.044 | 4.14E-74 |
| SPP1 | ENSG00000118785.13 | 75.378 | 8.524 | 3.004 | 2.34E-15 |
| STMN1 | ENSG00000117632.20 | 56.668 | 6.28 | 2.986 | 1.65E-75 |
| CDC20 | ENSG00000117399.13 | 9.32 | 0.31 | 2.978 | 3.93E-63 |
| LYZ | ENSG00000090382.6 | 81.413 | 9.505 | 2.972 | 1.14E-24 |
| FTH1P7 | ENSG00000232187.1 | 25.5 | 2.38 | 2.971 | 2.02E-72 |
| SPINK1 | ENSG00000164266.10 | 187.155 | 23.855 | 2.92 | 1.47E-06 |
| TOP2A | ENSG00000131747.14 | 9.54 | 0.405 | 2.907 | 3.35E-65 |
| THY1 | ENSG00000154096.13 | 20.42 | 1.995 | 2.838 | 1.97E-62 |
| RP5-940J5.9 | ENSG00000269968.1 | 20.359 | 2.015 | 2.825 | 1.22E-06 |
| CD24 | ENSG00000272398.5 | 50.85 | 6.745 | 2.743 | 5.43E-13 |
| SFN | ENSG00000175793.11 | 9.74 | 0.605 | 2.742 | 1.61E-31 |
| PDZK1IP1 | ENSG00000162366.7 | 15.74 | 1.505 | 2.74 | 1.12E-16 |
| LINC00152 | ENSG00000222041.10 | 35.26 | 4.54 | 2.711 | 1.71E-59 |
| TSPAN8 | ENSG00000127324.8 | 52.008 | 7.16 | 2.7 | 2.31E-22 |
| RRM2 | ENSG00000171848.13 | 9.26 | 0.595 | 2.685 | 1.36E-50 |
| HSPB1P1 | ENSG00000236060.2 | 5.31 | 0 | 2.658 | 1.66E-49 |
| MIR4435-2HG | ENSG00000172965.14 | 33.1 | 4.45 | 2.645 | 4.40E-53 |
| ALG1L | ENSG00000189366.9 | 7.27 | 0.325 | 2.642 | 9.46E-43 |
| LCN2 | ENSG00000148346.11 | 38.101 | 5.525 | 2.583 | 1.58E-13 |
| CXCL10 | ENSG00000169245.5 | 11.72 | 1.145 | 2.568 | 3.36E-23 |
| CAPG | ENSG00000042493.15 | 26.6 | 3.725 | 2.546 | 7.53E-36 |
| TROAP | ENSG00000135451.12 | 6.69 | 0.325 | 2.537 | 3.39E-57 |
| UBE2T | ENSG00000077152.9 | 8.81 | 0.695 | 2.533 | 9.85E-75 |
| CD34 | ENSG00000174059.16 | 14.17 | 1.625 | 2.531 | 8.03E-94 |
| ZWINT | ENSG00000122952.16 | 9.69 | 0.865 | 2.519 | 7.03E-58 |
| VWF | ENSG00000110799.13 | 14.41 | 1.715 | 2.505 | 7.16E-57 |
| FTH1P20 | ENSG00000226564.1 | 13.07 | 1.48 | 2.504 | 1.81E-84 |
| MUC13 | ENSG00000173702.7 | 5.82 | 0.22 | 2.483 | 6.99E-25 |
| EEF1A2 | ENSG00000101210.10 | 7.09 | 0.45 | 2.48 | 9.75E-14 |
| NQO1 | ENSG00000181019.12 | 11.72 | 1.285 | 2.477 | 2.02E-29 |
| RP11-452N17.1 | ENSG00000277998.1 | 37.569 | 5.96 | 2.47 | 9.36E-52 |
| CENPF | ENSG00000117724.12 | 6.14 | 0.295 | 2.463 | 1.12E-53 |
| PRC1 | ENSG00000198901.13 | 10.68 | 1.12 | 2.462 | 3.32E-64 |
| CDK1 | ENSG00000170312.15 | 6.32 | 0.33 | 2.46 | 7.38E-61 |
| TK1 | ENSG00000167900.11 | 17.25 | 2.335 | 2.452 | 2.99E-58 |
| GBA | ENSG00000177628.15 | 73.48 | 12.61 | 2.452 | 2.46E-103 |
| RP11-334E6.12 | ENSG00000263873.1 | 6.87 | 0.46 | 2.43 | 1.24E-44 |
| RP5-890E16.4 | ENSG00000266341.1 | 4.32 | 0 | 2.411 | 2.20E-14 |
| IFI27 | ENSG00000165949.12 | 145.209 | 26.64 | 2.403 | 3.73E-19 |
| HLA-H | ENSG00000206341.7 | 36.931 | 6.175 | 2.402 | 1.66E-34 |
| HULC | ENSG00000251164.1 | 307.319 | 57.466 | 2.399 | 1.54E-20 |
| CENPM | ENSG00000100162.14 | 5.97 | 0.33 | 2.39 | 8.75E-58 |
| BIRC5 | ENSG00000089685.14 | 6.81 | 0.505 | 2.376 | 1.57E-54 |
| EPS8L3 | ENSG00000198758.10 | 4.76 | 0.13 | 2.35 | 2.06E-30 |
| E2F1 | ENSG00000101412.12 | 5.49 | 0.28 | 2.342 | 2.90E-57 |
| RBP7 | ENSG00000162444.11 | 17.259 | 2.635 | 2.329 | 3.32E-59 |
| COL4A1 | ENSG00000187498.14 | 26.299 | 4.465 | 2.321 | 3.62E-43 |
| BLVRA | ENSG00000106605.10 | 19.46 | 3.145 | 2.303 | 3.46E-46 |
| ROBO1 | ENSG00000169855.19 | 9.19 | 1.07 | 2.299 | 1.09E-32 |
| ST8SIA6-AS1 | ENSG00000204832.9 | 3.81 | 0 | 2.266 | 2.87E-27 |
| AC104534.3 | ENSG00000268083.5 | 5.81 | 0.42 | 2.262 | 6.03E-12 |
| LGALS4 | ENSG00000171747.8 | 224.27 | 46.12 | 2.257 | 2.48E-06 |
| PPIAP22 | ENSG00000198618.5 | 165.466 | 34.006 | 2.25 | 9.13E-79 |
| APOC2 | ENSG00000234906.8 | 2260.615 | 475.779 | 2.246 | 3.11E-13 |
| HNRNPCP2 | ENSG00000204253.4 | 7.96 | 0.895 | 2.241 | 1.00E-63 |
| HMGA1 | ENSG00000137309.19 | 36.319 | 6.9 | 2.24 | 8.96E-51 |
| FTH1P8 | ENSG00000219507.4 | 9.88 | 1.33 | 2.223 | 4.15E-60 |
| RP11-1143G9.4 | ENSG00000257764.2 | 17.64 | 3.005 | 2.219 | 4.02E-19 |
| MMP11 | ENSG00000099953.9 | 5.24 | 0.35 | 2.209 | 9.60E-39 |
| SPC24 | ENSG00000161888.11 | 5.15 | 0.33 | 2.209 | 5.18E-66 |
| NUDT1 | ENSG00000106268.15 | 15.59 | 2.595 | 2.206 | 2.77E-77 |
| RNASEH2A | ENSG00000104889.4 | 16.151 | 2.735 | 2.199 | 4.56E-77 |
| ACSM1 | ENSG00000166743.9 | 8.56 | 1.085 | 2.197 | 2.43E-16 |
| CTB-63M22.1 | ENSG00000229119.3 | 136.882 | 29.105 | 2.195 | 1.57E-20 |
| CCNB2 | ENSG00000157456.7 | 4.81 | 0.27 | 2.194 | 3.46E-57 |
| FABP5 | ENSG00000164687.10 | 16.66 | 2.88 | 2.186 | 1.76E-44 |
| HKDC1 | ENSG00000156510.12 | 8.11 | 1.01 | 2.18 | 3.33E-23 |
| TMEM150B | ENSG00000180061.9 | 6.44 | 0.645 | 2.177 | 3.44E-36 |
| ERICH5 | ENSG00000177459.10 | 18.92 | 3.41 | 2.175 | 1.43E-14 |
| MCM5 | ENSG00000100297.15 | 21.45 | 4.025 | 2.16 | 2.99E-56 |
| MCM2 | ENSG00000073111.13 | 6.75 | 0.735 | 2.159 | 1.21E-49 |
| GMNN | ENSG00000112312.9 | 31.111 | 6.19 | 2.159 | 2.77E-59 |
| TM4SF4 | ENSG00000169903.6 | 430.001 | 95.683 | 2.156 | 2.86E-18 |
| KIFC1 | ENSG00000237649.7 | 4.37 | 0.205 | 2.156 | 2.31E-55 |
| AC005255.3 | ENSG00000256210.3 | 3.45 | 0 | 2.154 | 6.77E-37 |
| RP11-667K14.4 | ENSG00000262533.1 | 6.33 | 0.655 | 2.147 | 5.25E-22 |
| S100A10 | ENSG00000197747.8 | 241.168 | 54.345 | 2.129 | 2.01E-41 |
| CKS1BP3 | ENSG00000268942.2 | 5.44 | 0.475 | 2.126 | 1.92E-51 |
| CENPW | ENSG00000203760.8 | 8.1 | 1.1 | 2.115 | 3.78E-62 |
| KIAA0101 | ENSG00000166803.10 | 9.86 | 1.51 | 2.113 | 9.70E-50 |
| HLA-A | ENSG00000206503.11 | 714.504 | 165.214 | 2.106 | 3.22E-46 |
| TYMS | ENSG00000176890.15 | 16.4 | 3.05 | 2.103 | 2.21E-48 |
| EIF5AP4 | ENSG00000234743.1 | 3.95 | 0.16 | 2.093 | 9.78E-37 |
| MYBL2 | ENSG00000101057.15 | 4.07 | 0.19 | 2.091 | 3.56E-43 |

TableS1 Top 100 highly expressed genes in tumor tissue.
