# Supplementary material for: Multi-Omics Integration: Predicting Progression and Optimizing Clinical Treatment of Hepatocellular Carcinoma Through Malignant-Cell-Related Genes
Source: Int J Mol Sci. 2025 Jun 26;26(13):6135. doi: 10.3390/ijms26136135 (PMC12249523; doi:10.3390/ijms26136135)
Supplement: Supplementary file 1 [file ijms-26-06135-s001.zip › 修改后的supplementary file/TableS2.docx]

| Gene |
| --- |
| RAN |
| HSPE1 |
| SNRPF |
| RPL17 |
| SMU1 |
| SNRPA1 |
| RRM1 |
| SNRPD1 |
| PSMA6 |
| PUF60 |
| RPS8 |
| PSMB3 |
| SARS1 |
| PLK1 |
| SF3B5 |
| RPL23 |
| RPS29 |
| PHF5A |
| PCNA |
| RPS19 |
| FAU |
| RPS20 |
| RPL3 |
| CDC27 |
| PSMA3 |
| RPS3A |
| RPS18 |
| RPL18A |
| RPS11 |
| SF1 |
| RPL8 |
| PRPF38A |
| POLR2L |
| RPL31 |
| KIF11 |
| SNU13 |
| RPL5 |
| CDC16 |
| RPL12 |
| LRR1 |
| PRPF19 |
| POLR3A |
| SRSF3 |
| SRSF2 |
| RPS3 |
| CDK1 |
| EIF4A3 |
| PRELID1 |
| RPS15A |
| RPL13 |
| RPL11 |
| RPA1 |
| RPS15 |
| PSMB4 |
| CDC45 |
| SNRNP200 |
| RPL37A |
| POLR2C |
| PSMA2 |
| POLR2B |
| LSM2 |
| SF3A3 |
| SRP54 |
| POLD1 |
| TOP2A |
| ESPL1 |
| EEF2 |
| ERH |
| SF3B3 |
| CCT3 |
| DHDDS |
| HSPA9 |
| ANKLE2 |
| PSMA1 |
| ATP6V0C |
| RPA3 |
| COPB1 |
| HNRNPK |
| RPL10A |
| PHB2 |
| NSF |
| PSMA7 |
| RPS23 |
| RPS12 |
| DONSON |
| DTL |
| RPL4 |
| RPL19 |
| DYNLRB1 |
| RPS4X |
| GINS2 |
| BANF1 |
| CCT4 |
| RRM2 |
| CDC23 |
| GINS1 |
| RPL9 |
| INTS11 |
| POLR2F |
| CDC7 |

TableS2 Genes with the lowest Gene effect of 100 in HCC cells.
