# Supplementary material for: Multi-Omics Integration: Predicting Progression and Optimizing Clinical Treatment of Hepatocellular Carcinoma Through Malignant-Cell-Related Genes
Source: Int J Mol Sci. 2025 Jun 26;26(13):6135. doi: 10.3390/ijms26136135 (PMC12249523; doi:10.3390/ijms26136135)
Supplement: Supplementary file 1 [file ijms-26-06135-s001.zip › 修改后的supplementary file/TableS3.docx]

| Abbreviation | PMID | Gene Category | Number of Genes | Application | Key Genes |
| --- | --- | --- | --- | --- | --- |
| PRGs | 34820376 | Pyroptosis & Ferroptosis / Programmed Cell Death | 3 | Prognosis, Immune Infiltration | GSDME, GPX4, SCAF11 |
| RRGs | 33828988 | Inflammatory Response / Immune Status | 8 | Prognosis, Immune Status | ADORA2B, MEP1A, P2RX4, SERPINE1, ITGA5, NOD2, RIPK2, SLC7A1 |
| CRPs | 36250008 | Metabolic Reprogramming / Immune (Potentially Cuproptosis-Related) | 5 | Prognosis, Potential Value in Immunotherapy | C7, MAGEA6, HK2, CYP26B1, EPO |
| RBMs | 37410140 | RNA Binding Proteins (RBPs) / Post-transcriptional Regulation | 4 | Prognosis | RBM8A, RBM19, RBM28, RBM45 |
| ARGs | 36769187 | Apoptosis / Anoikis / Tumor Invasion (Potentially Apoptosis-Related) | 5 | Prognosis | BAK1, SPP1, BSG, PBK, DAP3 |
| ICDs | 37382674 | Immunogenic Cell Death (ICD) / Immune Checkpoint | 3 | Outcomes Prediction, Response to TACE & Immunotherapy | DNASE1L3, KLRB1, LILRB1 |

TableS3 Comparison of Previously Published Prognostic/Predictive Models in Hepatocellular Carcinoma.
