# Supplementary material for: Multi-Omics Integration: Predicting Progression and Optimizing Clinical Treatment of Hepatocellular Carcinoma Through Malignant-Cell-Related Genes
Source: Int J Mol Sci. 2025 Jun 26;26(13):6135. doi: 10.3390/ijms26136135 (PMC12249523; doi:10.3390/ijms26136135)
Supplement: Supplementary file 1 [file ijms-26-06135-s001.zip › 修改后的supplementary file/TableS4.docx]

|  | F (5'-3') | R(5'-3') |
| --- | --- | --- |
| SRSF7 | CGGTACGGAGGAGAAACCAAG | AGCCACAAATCACCTTTCCATC |
| siSRSF7#1 | CCUCGACGAUCAAGAUCUAUC | UAGAUCUUGAUCGUCGAGGAG |
| siSRSF7#2 | GCUUCACUCAGAAGAUCUAGG | UAGAUCUUCUGAGUGAAGCUG |

TableS4 The siRNA and primer sequences.
